# Agro Active Potential of *Bacillus subtilis* PE7 against *Didymella bryoniae* (Auersw.), the Causal Agent of Gummy Stem Blight of *Cucumis melo*

**DOI:** 10.3390/microorganisms12081691

**Published:** 2024-08-16

**Authors:** Seo Kyoung Jeong, Seong Eun Han, Prabhakaran Vasantha-Srinivasan, Woo Jin Jung, Chaw Ei Htwe Maung, Kil Yong Kim

**Affiliations:** 1 Department of Plant Protection and Quarantine, Chonnam National University, Gwangju 61186, Republic of Korea; agrisun@korea.kr (S.K.J.); woojung@jnu.ac.kr (W.J.J.); 2 Department of Agricultural Chemistry, Environmentally-Friendly Agricultural Research Center, College of Agriculture and Life Sciences, Chonnam National University, Gwangju 61186, Republic of Korea; tjddms1160@naver.com; 3 Department of Applied Biology, College of Agriculture and Life Sciences, Chonnam National University, Gwangju 61186, Republic of Korea; vasanth.bmg@gmail.com

**Keywords:** microbial pesticides, volatile organic compounds, black rot, antifungal, melon

## Abstract

Microbial agents such as the *Bacillus* species are recognized for their role as biocontrol agents against various phytopathogens through the production of diverse bioactive compounds. This study evaluates the effectiveness of *Bacillus subtilis* PE7 in inhibiting the growth of *Didymella bryoniae*, the pathogen responsible for gummy stem blight (GSB) in cucurbits. Dual culture assays demonstrate significant antifungal activity of strain PE7 against *D. bryoniae*. Volatile organic compounds (VOCs) produced by strain PE7 effectively impede mycelial formation in *D. bryoniae*, resulting in a high inhibition rate. Light microscopy revealed that *D. bryoniae* hyphae exposed to VOCs exhibited abnormal morphology, including swelling and excessive branching. Supplementing a potato dextrose agar (PDA) medium with a 30% *B. subtilis* PE7 culture filtrate significantly decreased mycelial growth. Moreover, combining a 30% culture filtrate with half the recommended concentration of a chemical fungicide yielded a more potent antifungal effect than using the full fungicide concentration alone, inducing dense mycelial formation and irregular hyphal morphology in *D. bryoniae*. Strain PE7 was highly resilient and was able to survive in fungicide solutions. Additionally, *B. subtilis* PE7 enhanced the nutrient content, growth, and development of melon plants while mitigating the severity of GSB compared to fungicide and fertilizer treatments. These findings highlight *B. subtilis* PE7 as a promising biocontrol candidate for integrated disease management in crop production.

## 1. Introduction

Netted melon (*Cucumis melo* L. var. reticulatus Naud) is a member of the Cucurbitaceae family, distinguished by its netted rind and sweet, aromatic flesh [1]. This annual, sprawling vine features large, lobed leaves and tendrils that enable it to climb. Netted melons are globally significant due to their high nutritional value, sweet flavor, and versatility in culinary uses. They are a major agricultural product with substantial economic impact, contributing to the livelihoods of farmers and the agricultural economy. In addition, their popularity and high market demand across various regions make them a key commodity in international trade. They are rich in vitamins, antioxidants, and dietary fiber, making them a nutritious dietary addition. China is the largest producer of netted melons, supplying a substantial portion of the global market [2]. Melon production can be influenced by various factors, with insect pests and diseases playing a significant role in hampering productivity. Insect pests such as aphids, whiteflies, and thrips feed on plant sap, causing stunted growth and transmitting viral diseases that result in the distortion and discoloration of leaves [3].

Pathogen attacks also pose a significant obstacle to attaining high yields in this crucial fruit crop. Gummy stem blight (GSB), caused by *Didymella bryoniae* (Auersw.), significantly reduces melon yields and compromises the quality of the fruits [4,5]. The infection begins with the germination of spores produced by the pathogen *D. bryoniae*. Infection occurs through natural openings or wounds on plant parts, with subsequent colonization, lesion development, and spore production contributing to the spread of the disease, particularly under favorable environmental conditions [6]. Moisture and temperature significantly influence the germination, sporulation, and colonization of *D. bryoniae* conidia in plant tissue, as well as symptom development. The ideal conditions for infection occur within a relative humidity (RH) of 85% and a temperature range of 61–75 °F [7]. Rahman et al. [8] reported that the incidence of *D. bryoniae* can increase rapidly under warm and humid conditions in both greenhouses and natural fields, leading to substantial yield reductions ranging from 15% to 50%. Infected fruits may exhibit symptoms such as lesions, discoloration, and decay, making them less appealing to consumers [9,10]. These phytopathogenic fungi are widespread and have been reported in various countries across different continents, leading to significant yield losses by affecting fruit quality and yield, in addition to directly contributing to economic losses for watermelon growers [11]. GSB poses a serious threat to agriculture worldwide, because it can occur on or within seeds and transplants, facilitating its transmission across continents. Therefore, concerns have been raised regarding the global spread of this disease [12]. GSB management relies on fungicides [8], with chlorothalonil, mancozeb, azoxystrobin, and propiconazole being the most commonly used compounds in watermelon crops [13]. Seblani et al. [14] recently highlighted the importance of GSB management due to the lack of resistant cultivars and the significant ability of fungi to develop resistance to commercial fungicides. Given the growing consumer demand for pesticide-free products, alternative disease control methods, particularly biological control strategies, have recently garnered increasing attention [13,15,16]. 

Biologically active compounds from microbial antagonists are increasingly recognized as preferable and harmless alternatives to chemical pesticides for environmentally friendly plant disease management [17]. Members of the *Bacillus* genus play a significant role in suppressing plant diseases, leveraging their considerable potential to combat various phytopathogens. These bacteria employ a range of mechanisms, including volatile organic compounds, antibiotics, lytic enzymes, and the indirect activation of the plant’s native immune system, making them effective agents in disease control [18]. The Gram-positive bacterium *Bacillus subtilis* effectively suppresses plant diseases while simultaneously promoting plant growth [19,20]. *B. subtilis* employs diverse mechanisms to compete with plant pathogens, exerting antagonistic activity on fungal strains by restricting their resources and impeding their spatial development [21]. Additionally, *B. subtilis* synthesizes a variety of antimicrobial compounds, including antibiotics, peptides, and secondary metabolites, which play a crucial role in inhibiting the growth and development of pathogens [22,23]. Furthermore, strains of *B. subtilis* activate the plant’s innate immune system, enhancing its resistance to pathogenic attacks. This systemic resistance helps prevent and manage severe diseases in melons, such as *Fusarium* wilt and GSB [24,25]. Despite the widespread recognition of *B. subtilis* as a bioactive strain with plant growth–promoting properties, no previous studies have explored the interactions of the specific strain *B. subtilis* PE7 and its associated volatile organic compounds (VOCs) with commercial fungicides, and how they affect *D. bryoniae* growth. Given the potential advantages of *B. subtilis* as a promising agro-active agent for crop growth and disease management, this study sought to (1) investigate the antagonistic activity of *B. subtilis* PE7 against *D. bryoniae*, (2) examine the antifungal effects of VOCs released by *B. subtilis* PE7 on the growth of *D. bryoniae*, (3) evaluate the antifungal effect of active metabolites of *B. subtilis* PE7 and its potential synergistic interaction with fungicides on the growth of *D. bryoniae*, (4) determine the effect of the fungicide on the survival of *B. subtilis* PE7, and (5) assess whether *B. subtilis* PE7 promotes the growth of melon plants. 

## 2. Materials and Methods

### 2.1. Culture Conditions and Microorganisms

The microbial strain *B. subtilis* PE7 (Acc. No. 92549P) from the Korean Agricultural Culture Collection (KACC) employed in this study was initially isolated from cabbage kimchi. *B. subtilis* was subcultured on a tryptone soy agar (TSA) medium at 30 °C, and strain PE7 was inoculated in a tryptone soy broth (TSB) medium for 2 days in a shaking incubator. The broth culture was then preserved at −80 °C after being mixed with a sterile 50% glycerol solution for subsequent experiments. The fungal pathogen *D. bryoniae* (Acc. No. KACC 40669) was obtained from the KACC and subcultured on a potato dextrose agar (PDA) medium at 25 °C for 7 days.

### 2.2. Dual Culture and Mycelial Growth Inhibition Assay

A mycelial plug (5 mm diameter) obtained from a 6-day-old colony of *D. bryoniae* was placed on one side of a sterile PDA plate. Simultaneously, a colony of *B. subtilis* PE7 was streaked on the opposite side of the same medium. The PDA medium inoculated solely with *D. bryoniae* was employed as a control. The experiment was conducted in triplicate. The plates were then incubated at 25 °C for 7 days, and mycelial growth inhibition was calculated using Formula (1)

Mycelial growth inhibition (%) = R1 − R2/R1 × 100(1)

where R1 represents the radius of the *D. bryoniae* colony in the control plate, whereas R2 is the radius of the *D. bryoniae* colony in the dual-culture plate. This experiment was adapted following the protocol described by Maung et al. [20].

### 2.3. Inhibitory Activity of B. subtilis PE7 VOCs on D. bryoniae Growth

The antifungal activity of VOCs was assessed as described by Maung et al. [26]. A 100 µL aliquot of a 1-day-old broth culture containing *B. subtilis* PE7 (10^8^ CFU mL^−1^) was spread onto a TSA medium, after which a 5 mm diameter mycelial plug from a 6-day-old *D. bryoniae* colony was placed at the center of the PDA medium. The bacterial and fungal plates were sealed together with parafilm, while a control TSA plate spread with 100 µL sterile distilled water was similarly sealed with the fungal plate. Three replicate plates were used for each experimental condition, and all plates were incubated at 25 °C for 7 days. The effect of *B. subtilis* PE7 VOCs on *D. bryoniae* mycelial growth was calculated using Formula (2)

Mycelial growth inhibition (%) = (D − d)/D × 100(2)

where D represents the diameter of the *D. bryoniae* colony in the control plate, whereas d denotes the diameter of the *D. bryoniae* colony in the VOCs-affected plate. Small pieces of mycelia from both the control and VOCs-affected plates were examined under a light microscope to observe the hyphal morphology of *D. bryoniae*. 

### 2.4. Effect of Chemical Fungicide on B. subtilis PE7 Growth

A single colony of *B. subtilis* PE7 was preinoculated in 100 mL of a pink–brown broth (PBB) medium and incubated at 30 °C and 130 rpm for 3 days. The preinoculated broth culture (500 µL, 10^7^ CFU mL^−1^) was then transferred into 500 mL of fresh PBB medium and incubated at 30 °C and 130 rpm for an additional 2 days. Subsequently, 100 mL of the broth culture of strain PE7 (10^8^ CFU mL^−1^) was transferred to a sterile flask containing 100 mL of sterile distilled water, and a chemical fungicide (50% trifloxystrobin) was added to achieve a final concentration of 250 ppm. A control was established using the diluted broth culture without the addition of the chemical fungicide. The flasks were incubated at 30 °C in a shaking incubator at 130 rpm, and samples were collected from each flask at 0, 1, 3, 5, and 7 days after incubation (DAI). Cell numbers in each sample were determined via the serial dilution and plate count method by spreading the cultures on the TSA medium.

### 2.5. In Vitro Examination of D. bryoniae Growth Inhibition by B. subtilis PE7

An individual colony of *B. subtilis* PE7 was preinoculated in a pink–brown broth (PBB) medium at 30 °C and 130 rpm for 2 days. Subsequently, 500 µL of the preinoculated broth culture of *B. subtilis* PE7 (10^7^ CFU mL^−1^) was reinoculated into a flask containing 500 mL of fresh PBB medium and incubated at 30 °C and 130 rpm in a shaking incubator for 7 days. After incubation, the broth culture was centrifuged at 12,000 rpm and 4 °C for 15 min. The resulting supernatant was collected and filtered using a 0.2 µm syringe filter. This culture filtrate (CF) was used for antifungal assays against *D. bryoniae*. The CF of strain PE7 was blended with a sterile-cool PDA medium in a flask to achieve a final concentration of 30% (30% CF). Separately, 125 ppm of the chemical fungicide (50% trifloxystrobin) was added to another flask containing a sterile-cool PDA medium with 30% CF, resulting in a concentration of 30% CF + 50% F. The PDA medium mixed with each fungicide concentration (125 ppm and 250 ppm) was denoted as 50% F and 100% F, respectively. Here, 100% F represented the recommended concentration of the chemical fungicide (250 ppm of 50% trifloxystrobin), and 50% F indicated half of the recommended concentration (125 ppm of 50% trifloxystrobin). The PDA medium without the addition of culture filtrate or fungicide served as the control, with triplicate plates employed for each treatment. Subsequently, a 5 mm diameter mycelial plug from a 6-day-old colony of *D. bryoniae* was placed at the center of each PDA plate for each treatment, and the plates were incubated at 25 °C for 5 days. Mycelial growth inhibition was calculated as described in Section 2.3.

### 2.6. Preparation of D. bryoniae Conidial Suspension for Pot Experiments

Oriental melons were surface-sterilized with 1% NaOCl for 3 min, followed by 70% ethanol for 3 min, and then rinsed three times with double-distilled water. A sterile cork borer was used to create two wounds (approximately 2.5 mm in depth) spaced 5 cm apart on the surface of each melon. Subsequently, a mycelial plug of *D. bryoniae* was inoculated into each wound. The melons were then placed in plastic boxes and incubated at 25 °C with a 12-h photoperiod and 88% RH for 12 days. The melon tissues containing black fruiting bodies of *D. bryoniae* were homogenized with sterile distilled water, and the resulting conidia were filtered through two layers of sterile cheesecloth. The concentration of conidia in the suspension was determined by counting using a hemocytometer under a light microscope and then adjusted with sterile distilled water to achieve a 10^5^ conidia mL^−1^ density.

### 2.7. In Vivo Pot Experiment 

*Cucumis melo* L. var. reticulatus Naud seeds were initially sown in organic bed soil for 3 weeks. Upon reaching the two-leaf stage, each melon seedling was transplanted into pots containing a soil mixture (soil; sand: vermiculite, 1:1:1 *v*/*v*/*v*). The experiment included three treatments as follows: fertilizer as the control (Con), fertilizer with the recommended concentration of fungicide (FF), and *B. subtilis* PE7 culture (PE7). Each treatment was replicated three times, with five melon plants per replicate. Equivalent amounts of fertilizer used in the PBB medium were prepared for soil drenching in the Con and FF treatments. For the FF treatment, the recommended concentration (250 ppm) of fungicide (50% trifloxystrobin) was applied via foliar spraying according to label instructions. For the PE7 treatment, we employed a 7-day-old broth culture of *B. subtilis* PE7 (10^7^ CFU mL^−1^) inoculated in the PBB medium at 30 °C and 130 rpm. At 7 days post-transplantation, 25 mL of each treatment was applied to the melon plants via soil drenching as the first and second treatments at weekly intervals. For the PE7 and FF treatments, foliar spraying was conducted with 15 mL of bacterial culture and 15 mL of fungicide solution, respectively. For the third treatment, 50 mL was applied via soil drenching and 25 mL via foliar spraying of each treatment. Subsequently, for the fourth, fifth, sixth, and seventh treatments, 65 mL of each treatment was soil drenched, and 35 mL of bacterial culture or fungicide solution was sprayed on the leaves. During the sixth and seventh treatments, a total of 10 mL of conidial suspension (1 × 10^5^ spore mL^−1^) was thoroughly sprayed on the leaves of each melon plant. At 16 days after pathogen inoculation, the disease severity of melon leaves was assessed using a rating scale modified from Hassan et al. [27]: 1 = 0% of leaf area infected, 2 = 1–5% of leaf area infected, 3 = 6–10% of leaf area infected, 4 = 11–30% of leaf area infected, 5 = 31–50% of leaf area infected, and 6 = 51–100% of leaf area infected. The percent disease index (PDI) was calculated using Equation (3), as described by Rahman et al. [8]

Percent disease index (PDI) = ∑ (class rating × class frequency)/Total number of leaves counted × maximum rating value × 100 (3)

The plant growth analyses included measurements of leaf number, leaf area, and lengths, as well as the fresh and dry weights of shoots and roots. Dry weights were determined after oven-drying at 70 °C for 3 days. Additionally, the chlorophyll content of melon leaves was measured at weekly intervals from the fourth to the seventh week after transplantation using a chlorophyll meter (SPAD 502-plus).

### 2.8. Quantification of B. subtilis PE7 in Pot Soil

The potting soil from melon plants subjected to the PE7 treatment was collected and air-dried under ambient room conditions. Subsequently, 1 g of soil was blended with 9 mL of sterile distilled water and placed in a shaker for 15 min. The cell numbers of strain PE7 were determined through a serial dilution and plate count method on the TSA medium. The colonies of strain PE7 were counted following incubation at 30 °C for 2 days.

### 2.9. Nutrient Analysis of Melon Plants

The oven-dried melon plants from each treatment group (Con, FF, PE7) were ground into a fine powder using a grinder. The dry powders from three plants in each treatment were then utilized for nutrient analysis. Essential nutrients, including phosphorus (P), potassium (K), calcium (Ca), magnesium (Mg), sulfur (S), boron (B), copper (Cu), iron (Fe), manganese (Mn), and molybdenum (Mo), were quantified by adding 0.5 g of each sample, 1 mL of hydrogen peroxide, and 9 mL of nitric acid into a Kjeldahl digestion tube. The mixtures were then allowed to react overnight. The samples were then digested by heating at 100 °C for 30 min, 150 °C for 30 min, and 200 °C for 1 h using a digestion block. After digestion, the samples were diluted with double-distilled water (DDH_2_O) to a final volume of 100 mL, filtered through two layers of Whatman Grade 6 filter papers, and analyzed using an inductively coupled plasma optical emission spectrometer (ICP-OES). To determine the nitrogen (N) content of the melon plants, 50 mg of each dried sample was placed into a titanium capsule and analyzed using a vario MAX cube elemental analyzer (ELEMENTAR, Langenselbold, Germany).

### 2.10. Data Analysis

The data on mycelial growth inhibition caused by the culture filtrate of *B. subtilis* PE7, as well as the growth parameters and nutrient contents of melon plants, were subjected to analysis of variance (ANOVA) using SAS version 9.4. Mean comparisons were conducted using the least significant difference (LSD) test at a significance level of *p* < 0.05.

## 3. Results

### 3.1. Antagonistic Activity of B. subtilis PE7 against D. bryoniae

**Figure 1:**
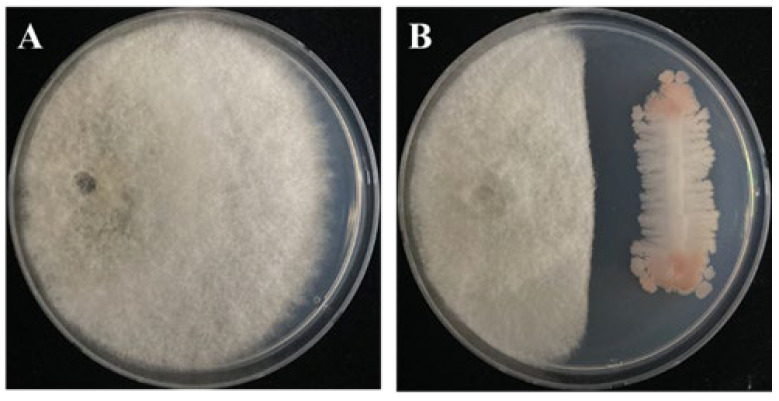
Antifungal activity of *B. subtilis* PE7 against *D. bryoniae* on the PDA medium. (**A**) Control plate with *D. bryoniae* only. (**B**) Plate with *B. subtilis* PE7 and *D. bryoniae* co-culture showing inhibition at 7 days of incubation.

The dual culture assay results revealed that *B. subtilis* PE7 significantly inhibited the growth of the pathogen *D. bryoniae* compared to the control (Figure 1A). At 7 days of incubation, the inhibition rate was 57.14 ± 1.2%, as shown in Figure 1B. 

### 3.2. Inhibitory Effect of B. subtilis PE7 VOCs on D. bryoniae

**Figure 2:**
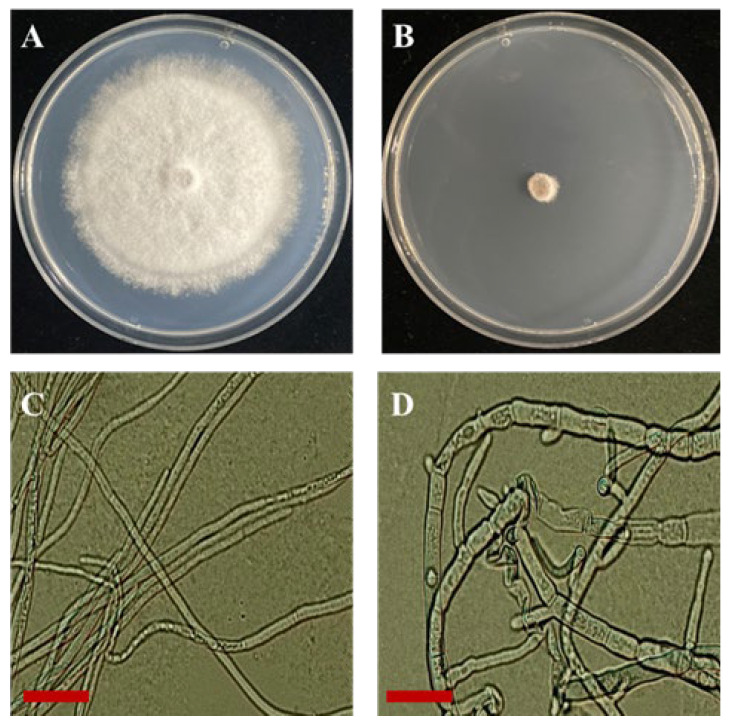
Inhibitory effect of *B. subtilis* PE7 VOCs on *D. bryoniae*. (**A**) Control plate with *D. bryoniae* only. (**B**) Plate showing inhibition of *D. bryoniae* by VOCs from *B. subtilis* PE7. (**C**) Normal hyphal structure of *D. bryoniae* in the control. (**D**) Twisted and swollen hyphal structure of *D. bryoniae* affected by VOCs from *B. subtilis* PE7 (scale bar = 25 µm).

The mycelial growth of the fungal pathogen *D. bryoniae* was markedly influenced by the VOCs of *B. subtilis* PE7, resulting in an inhibition rate of 85.4 ± 3.10% (Figure 2B) compared to the control (Figure 2A). Examination under light microscopy revealed that the hyphae affected by VOCs were intricately twisted and swollen (Figure 2D), whereas the control hyphae displayed normal and straight structures (Figure 2C).

### 3.3. Influence of Chemical Fungicide on the Survival of B. subtilis PE7

**Figure 3:**
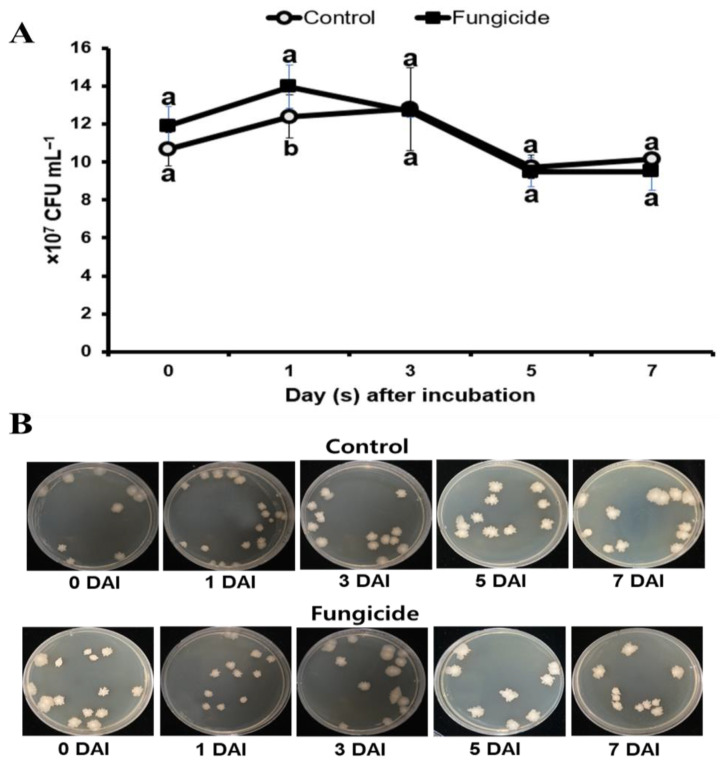
Effect of chemical fungicide (50% trifloxystrobin, 250 ppm) on the survival of *B. subtilis* PE7. (**A**) Growth rate comparison between the control and fungicide-treated *B. subtilis* PE7 at a concentration of 250 ppm. Different letters indicate significant differences between control and fungicide treatment according to Student’s *t*-test (*p* < 0.05). (**B**) Consistent colony numbers of strain PE7 from 0 to 7 days after incubation in both control and fungicide treatments.

The growth of *B. subtilis* PE7 was examined following treatment with the commercial fungicide over a 7-day incubation period (Figure 3). The presence of fungicide had no remarkable impact on the growth of *B. subtilis* PE7, because the growth rate remained steady compared to the control even at a 250 ppm concentration (Figure 3A). No significant difference was observed between the control and fungicide treatment after 7 days of incubation. Strain PE7 displayed consistent growth and maintained similar colony numbers from 0 to 7 days after incubation, as illustrated in Figure 3B.

### 3.4. B. subtilis PE7 Culture Filtrate and Fungicide Co-Exposure for Targeted Suppression of D. bryoniae

**Figure 4:**
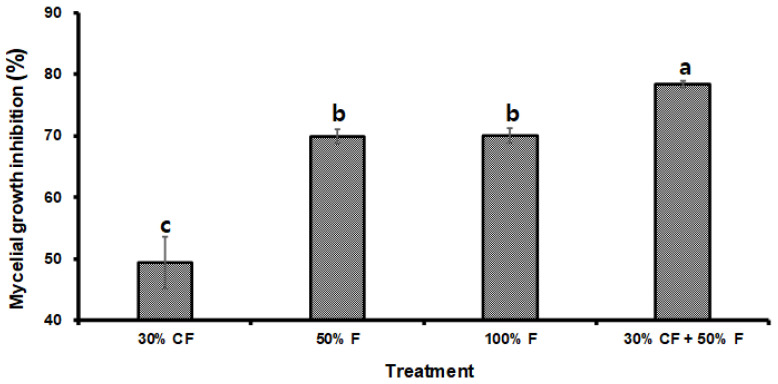
Inhibition of *D. bryoniae* mycelial growth treated with varying concentrations of *B. subtilis* PE7 culture filtrate (CF) and fungicide (F), as well as their combination (100% F and 50% F correspond to 250 ppm and 125 ppm of 50% trifloxystrobin, respectively). The data were presented as the mean ± standard deviation of three replicates. Different letters in the bar graph indicate significant differences between treatments, as determined by the least significant difference (LSD) test at a *p* < 0.05 significance level.

The mycelial growth of *D. bryoniae* was notably inhibited after treatment with different concentrations of *B. subtilis* PE7 culture filtrate (CF) and a commercial fungicide (F). The highest inhibition percentage occurred with the combined treatment of 30% CF and 50% F, which was statistically significant compared to other treatments. Furthermore, there was no significant difference in mycelial inhibition rate between the 50% F and 100% F treatments (*p* < 0.05). Notably, the least mycelial growth inhibition was observed in the treatment with 30% CF alone (Figure 4).

### 3.5. Effects of B. subtilis PE7 CF and Fungicide on D. bryoniae Hyphal Morphology

**Figure 5:**
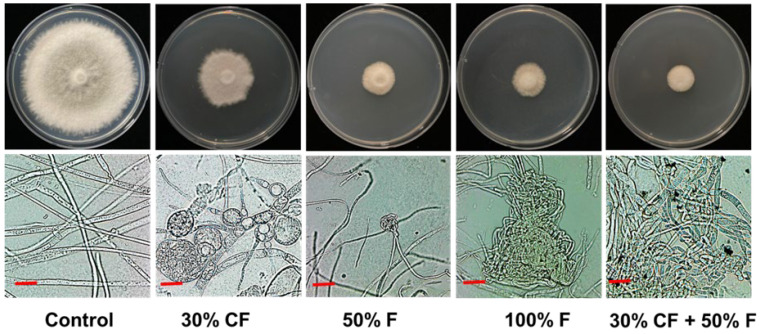
Comparison of abnormal mycelial growth and deformed hyphal morphology of *D. bryoniae*, characterized by bulbous or condensed structures, in treatments with *B. subtilis* PE7 culture filtrate (CF), fungicide (F), and their combination, relative to the control displaying normal morphology (scale bar = 25 µm).

The mycelial growth was significantly inhibited in both the CF and F treatments compared to the control, which exhibited normal mycelial development. The highest inhibition occurred with the combined treatment (30% CF + 50% F) compared to other treatments (30% F, 50% F, and 100% F). Microscopic examination of the hyphal morphology of the fungal pathogen *D. bryoniae* revealed abnormal expansion and alterations in the morphology of *D. bryoniae* treated with fungicide and CF. Notably, more severe damage characterized by twisting, excessive branching, bulging, and irregular twisting was observed in the treatment with 30% CF and the combined treatment (30% CF + 50% F) compared to the other fungicide treatments (Figure 5).

### 3.6. Effect of B. subtilis PE7 Culture on Melon Plant Growth

**Figure 6:**
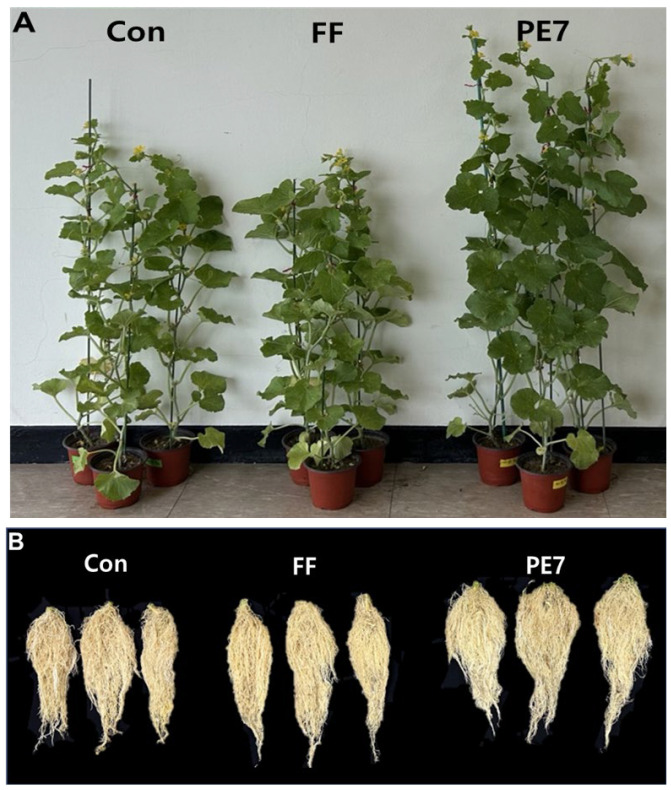
Influence of *B. subtilis* PE7 culture on the growth of melon plants. (**A**) Comparison of shoot length after 16 days postinoculation of *D. bryoniae*. (**B**) Comparison of fresh root weight. Con: fertilizer only; FF: fertilizer combined with fungicide; PE7: Broth culture of *B. subtilis* PE7.

**Figure 7:**
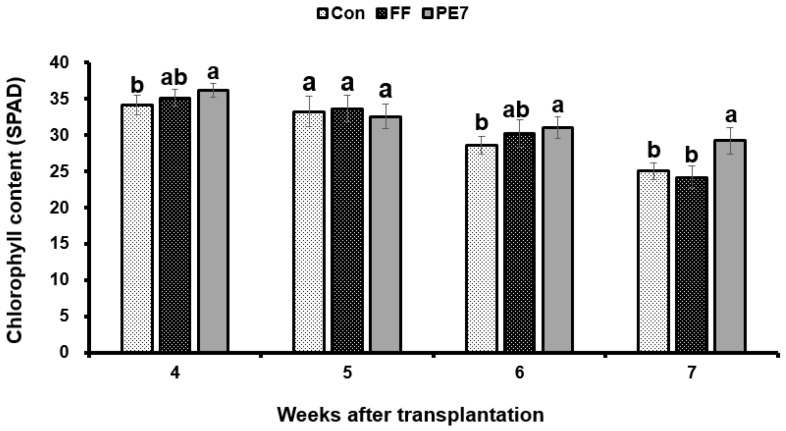
Chlorophyll content of melon leaves of different treatments at different weeks (4–7) post-transplantation. Con: fertilizer only; FF: fertilizer combined with fungicide; PE7: Broth culture of *B. subtilis* PE7. Different letters above each bar (grouped by post-transplantation week) indicate significant differences between treatments, as determined by the least significant difference (LSD) test at a *p* < 0.05 significance level.

The effects of different treatments on the growth of melon plants under potted conditions were evaluated over a 16-day period after *D. bryoniae* inoculation. Analysis of the results indicated enhanced plant growth, particularly in terms of shoot length (108.87 ± 5 cm) in the PE7 broth culture treatment compared to both the FF treatment (82.7 ± 5 cm) and the control (86.02 ± 1 cm). Notably, no significant difference in melon plant growth was observed between the FF treatment and the control, as depicted in Figure 6A. Moreover, the fresh weight of shoots also increased in the PE7 treatment (94.2 ± 4 g) compared to FF and the control (Table 1). Similarly, root length in melon plants showed an increase in the PE7 treatment (24.98 ± 1 cm) compared to the FF treatment (23.72 ± 0.7 cm) and the control (21.4 ± 1 cm). Fresh root weight was significantly increased in the PE7 treatment group (19.62 ± 4 g) compared to FF and the control (Figure 6B). Moreover, we observed an increase in the leaf number (24.8 ± 0.9) and leaf area (1388.6 cm^2^) in the PE7 treatment (Table 1). Furthermore, the chlorophyll content of melon plants increased considerably following treatment with the PE7 broth culture over a 4-week period post-transplantation compared to the control. However, during the initial 3 weeks post-transplantation, there was no significant disparity in chlorophyll content between the PE7 and FF treatments, with significance emerging only in the fourth week post-transplantation (Figure 7).

### 3.7. Effects of B. subtilis PE7 Culture and Fungicide on the Disease Severity of D. bryoniae-Infected Melon Plants

**Figure 8:**
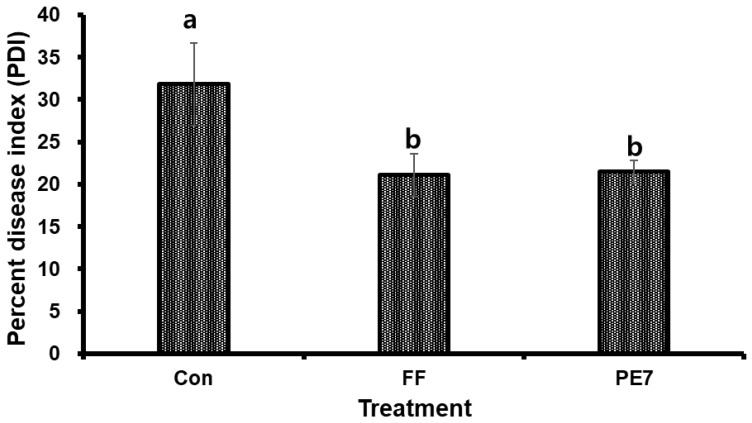
Reduction in disease severity of GSB in melon by *B. subtilis* PE7 culture and fungicide compared to the control at 16 days after *D. bryoniae* inoculation. Con: fertilizer only; FF: fertilizer combined with fungicide; PE7: Broth culture of *B. subtilis* PE7. Different letters in the bar graph indicate significant differences between treatments, as determined by the least significant difference (LSD) test at a *p* < 0.05 significance level.

The PDI of GSB was assessed across the following three distinct treatments: Con, FF, and PE7. The highest disease index was observed in the control group, whereas both the FF and PE7 treatments exhibited reduced disease severity. However, there was no statistically significant difference in disease severity between the PE7 and FF treatments 16 days following inoculation with *D. bryoniae* (Figure 8).

### 3.8. Nutrient Analysis of Melon Plants 

Compared to the control group (Con), melon plants treated with the broth culture of *B. subtilis* PE7 (PE7) exhibited significantly higher levels of major macronutrients, as detailed in Table 2. Specifically, the PE7-treated plants exhibited notable increases in nitrogen (N) at 199.23 mg plant^−1^, potassium (K) at 488.27 mg plant^−1^, magnesium (Mg) at 98.76 mg plant^−1^, and calcium (Ca) at 328.86 mg plant^−1^. However, there were no significant differences observed in macronutrient levels (P, K, Mg, and Ca) between the control and PE7-treated groups. Furthermore, the PE7 treatment resulted in elevated levels of micronutrients in melon plants compared to both the Con and FF treatments. Notably, the concentrations of manganese (Mn), boron (B), copper (Cu), iron (Fe), and zinc (Zn) increased to 7.56, 344.34, 1450.49, 30.82, and 0.50 mg plant^−1^, respectively, in the PE7-treated plants. However, there were no significant differences in micronutrient levels (Cu and Fe) between the FF and PE7 treatments. Additionally, the concentration of molybdenum (Mo) was found to be lowest in the PE7-treated plants at 4.07 mg plant^−1^ compared to the plants in the Con group at 7.25 mg plant^−1^ and the FF treatment at 6.43 mg plant^−1^.

## 4. Discussion

*Bacillus subtilis* strains are known for their ability to suppress plant pathogens through various antagonistic actions, including the production of antifungal compounds [28,29], outcompeting the pathogens for nutrients and space [21,30], inducing systemic resistance in plants by priming them to defend against pathogen attacks [31,32,33], forming biofilms on plant surfaces that create physical barriers preventing pathogen attachment and colonization [34,35], and producing enzymes such as chitinases and proteases that directly inhibit fungal growth and disrupt the pathogen’s lifecycle [36,37]. The dual culture methodology facilitates precise quantification of the antagonistic impact exerted by *Bacillus* strains against fungal pathogens. This is achieved by assessing key growth parameters, including radial growth inhibition, alterations in hyphal morphology, and reduction in biomass [38,39]. Our findings revealed that *B. subtilis* PE7 inhibits the growth of *D. bryoniae*, the causal agent of GSB, following a 7-day exposure period.

Grahovac et al. [40] recently reported that various strains of *B. subtilis* can generate a wide spectrum of VOCs characterized by diverse chemical compositions and biological properties, exhibiting the capacity to hinder the growth of fungal pathogens either through direct effects on fungal cells or by interfering with their physiological processes. Our findings were highly consistent with these observations. Specifically, VOCs emitted by *B. subtilis* significantly inhibited the mycelial growth of *D. bryoniae*. Al-Mutar et al. [41] also reported that extracellular lipopeptides and VOCs emitted from *B. subtilis* strain DHA41 exhibited strong antifungal activity against *D. bryoniae*, *F. oxysporum*, *F. graminearum*, and *Sclerotinia sclerotiorum*. Similarly, VOCs produced by *B. amyloliquefaciens* L3, including 2-ethyl-1-hexanol, 2-heptanone, and 2-nonanone, exhibited complete inhibition of *F. oxysporum* mycelial growth [6]. Leveraging the biocontrol potential of *B. subtilis* VOCs offers a sustainable and eco-friendly basis for managing fungal diseases in agriculture. The use of VOCs as natural fungicides can reduce the reliance on chemical pesticides and promote environmentally friendly crop protection practices. Waghunde et al. [42] reported that VOCs released by *B. subtilis* can lead to branching or fragmentation of hyphae in fungal pathogens, resulting in the development of irregular structures and impaired hyphal growth. Similarly, upon exposure to VOCs from *B. subtilis* PE7, *D. bryoniae* displayed distorted hyphal structures characterized by swelling and twisting. Ahmed et al. [43] reported that *B. amyloliquefaciens* WS-10 produced bioactive compounds with strong antifungal properties against various pathogens. The study emphasized the role of VOCs in disrupting the cell walls of fungal pathogens, thereby inhibiting their growth.

The potential synergism between commercial fungicides and *B. subtilis* is a crucial consideration in integrated pest management strategies. Therefore, assessing the potential interactions between fungicides and beneficial bacteria is critical to avoid negative impacts on biocontrol efficacy [44]. Certain chemical fungicides such as tebuconazole can have antagonistic effects on *B. subtilis* when applied at high rates, inhibiting its growth or activity and creating unfavorable conditions for its survival and proliferation, thereby potentially compromising its biocontrol efficacy [45]. The influence of chemical fungicides on the survival or synergistic interactions of *B. subtilis* is highly complex, and understanding the dynamics between fungicides and *B. subtilis* is essential for optimizing biocontrol strategies and promoting sustainable agriculture practices. Our findings indicate a consistent growth rate in *B. subtilis* PE7 when exposed to trifloxystrobin, maintaining colony numbers similar to those observed in the control group. This result aligns with those of Kondoh et al. [46], who reported that the chemical pesticide flutolanil had no remarkable effect on the growth of *B. subtilis* RB14-C. These findings support the potential for the co-administration of biocontrol agents with chemical fungicides, because bacterial survival did not appear to be negatively affected by the fungicide.

Synergistic interactions between *Bacillus* species and chemical fungicides in controlling fungal pathogens can enhance disease management strategies, including complementary modes of action [47]. In our study, treatment with 30% CF of strain PE7 significantly inhibited the growth of *D. bryoniae*. This inhibition is likely due to active metabolites accumulated in the CF of strain PE7. Numerous studies have shown that *Bacillus* species produce a variety of antifungal substances, including cyclic lipopeptides, polypeptides, proteins, and non-peptides, which serve as their primary defense against phytopathogenic microorganisms. Among these antimicrobial compounds, cyclic lipopeptides released by *B. subtilis* such as iturins, bacillomycins, fengycins, and surfactins are well-known for their inhibitory effects on the growth of phytopathogenic fungi by disturbing cell membranes, inducing osmotic imbalance, and leading to cell death [48,49]. Noh et al. [50] demonstrated that exposure to a CF of *B. subtilis* NM4 reduced the mycelial growth of *Alternaria solani* and led to hyphal deformations and abnormal bulbous morphology, which was attributed to the presence of surfactins and fengycins in the CF. Similarly, the bulging and severe swelling in the hyphal structures of *D. bryoniae* observed in the 30% CF treatment could be attributed to the action of these lipopeptides or related compounds. Moreover, our findings demonstrated that combining 30% CF of *B. subtilis* PE7 with 50% commercial fungicide resulted in the highest inhibition rate against *D. bryoniae*, surpassing the outcomes of individual treatments and the control group. This suggests synergism between the fungicide and strain PE7 metabolites, enhancing the growth inhibition of the fungal pathogen. By leveraging the biocontrol capabilities of *Bacillus* species alongside chemical fungicides, our current reliance on fungicides can be markedly decreased while maintaining effective disease management. This integrated approach can help minimize the use of synthetic chemicals while achieving sustainable disease control outcomes [51]. For instance, combining the chemical fungicide carbendazim 12% + mancozeb 63% with the bio-agent *Trichoderma harzianum* significantly reduced Fusarium wilt disease (21.72%) compared to the control (64.2%) [52]. Synergistic interactions can result in a broader spectrum of activity, increased pathogen inhibition, and enhanced plant protection against fungal pathogens [53]. *Bacillus subtilis* produces antifungal compounds that can inhibit fungal growth, including the elongation of hyphae [39]. When combined with chemical fungicides, which also inhibit hyphal growth, the overall impact on fungal morphology can be enhanced, leading to reduced hyphal length and branching [53,54]. Therefore, the synergistic interactions between antimicrobial metabolites produced by *B. subtilis* PE7 and chemical fungicides (30% CF + 50% F) result in more pronounced effects on fungal hyphal morphology than either treatment alone. This combined approach can provide enhanced control of fungal pathogens by disrupting hyphal growth and structure, thereby impeding the progression of fungal infections and reducing disease severity in plants [55].

In our pot experiment, the soil drench and foliar application of *B. subtilis* PE7 to melon plants significantly reduced the disease severity of GSB compared to the control. The efficacy of strain PE7 was comparable to that of the fungicide treatment. This outcome may be attributed to the cells and metabolites of strain PE7 disrupting the conidial germination and developmental processes of *D. bryoniae*, thereby reducing GSB in melon plants. Severe infections of GSB can weaken plants, reduce photosynthetic activity, and impair nutrient uptake, leading to decreased growth and yield. Additionally, gummy exudate from stem cankers can attract secondary pathogens, further compromising plant health [14]. The production of antimicrobial compounds and the alteration of microbial populations by *B. subtilis* can create an unfavorable environment for pathogen survival and proliferation, leading to decreased disease severity in plants [56]. A similar study by Sayago et al. [57] revealed that the administration of *B. subtilis* ALBA01 resulted in a lower PDI with a higher biocontrol level (>50%) against the soil-borne pathogen *Setophoma terrestris* in onions. Furthermore, *B. subtilis* B579 exhibited a remarkable 73% reduction in *F. oxysporum* disease incidence in *Cucumis sativus* (cucumber) plants [58]. 

*Bacillus subtilis* is known for its plant growth–promoting properties, including the production of substances such as indole-3-acetic acid (IAA) and siderophores [18,59]. Our previous studies have shown that inoculation with *B. subtilis* PE7 culture significantly enhances the overall growth of melon and tomato plants. Additionally, the presence of IAA in the PE7 culture positively affects root density and promotes nutrient acquisition, increasing both macro- and micronutrients in melon plants [60,61]. In vivo plant growth assays in the present study also revealed a notable enhancement in both root and shoot development, along with an increase in fresh leaf count in melon plants treated with PE7 strains, surpassing the effects observed with fungicide treatment and the control treated with fertilizer. Our nutrient analysis results further suggest that *B. subtilis* PE7 significantly improves the macro- and micronutrient content in *C. melo* compared to the control and FF treatment. Similar to our findings, microbial extracts from *Bacillus* LKE15 significantly improve the contents of major nutrients such as magnesium, potassium, and phosphorus in oriental melons [62]. Biologically active strains of *B. subtilis* employ a range of mechanisms that influence plant root and shoot growth. These mechanisms include the modulation of plant hormone production pathways such as cytokinins and gibberellins [63], nutrient solubilization in the soil [64], induction of stress tolerance mechanisms [65], triggering of the plant’s systemic resistance mechanisms [66], root colonization [67], regulation of gene expression in both the plant and the microbe through plant-microbe signaling [68,69,70], and promotion of enhanced root system architecture [71,72,73]. Furthermore, *B. subtilis* can enhance photosynthetic efficiency by optimizing photosystem functionality and augmenting light harvesting and utilization efficiency, thereby indirectly bolstering chlorophyll biosynthesis [74]. This is strongly supported by our current findings, which show a significant improvement in melon plant chlorophyll content following treatment with *B. subtilis* compared to fungicide application. In addition to direct antagonistic interactions, *B. subtilis* can exert indirect effects on pathogens by modulating the microbial community structure in the rhizosphere. In our study, the high numbers of viable cells of strain PE7 in the pot soil could be another reason for the growth enhancement of melon plants. Zhang et al. [75] demonstrated that microbial consortia involving *Bacillus* strains can significantly alter the rhizosphere bacterial community, enhancing plant resistance to pathogens. The study showed a substantial reduction in disease incidence when microbial consortia were used, suggesting a synergistic effect in biocontrol.

## 5. Conclusions

This study highlights the significant potential of *B. subtilis* PE7 as an effective biocontrol agent against *D. bryoniae*, the causative agent of GSB in cucurbits. By examining the impact of VOCs on the morphology of *D. bryoniae*, our findings demonstrated the potent antifungal properties of strain PE7. Additionally, our results revealed that PE7 culture filtrate, either alone or in combination with chemical fungicides, significantly inhibits *D. bryoniae* growth, offering a promising alternative to conventional fungicide treatments. The observed enhancement of the nutrient index and growth of melon plants, along with the reduction in disease severity, further underscores the potential of *B. subtilis* PE7 in integrated disease management strategies for sustainable crop production. This research provides valuable insights into harnessing microbial agents for effective disease control and crop health enhancement, paving the way for the development of innovative agricultural practices. In future studies, we will investigate the antifungal action of *B. subtilis* PE7 on different fungal pathogens and explore the active compounds responsible for the growth inhibition of these fungal pathogens. These compounds will be isolated using various chromatographic methods and identified through liquid chromatography–mass spectrometry (LC/MS) and nuclear magnetic resonance (NMR) analyses.

## Figures and Tables

**Table 1:** Plant growth parameters under different treatments at 16 days postinoculation with *D. bryoniae*. Con: fertilizer only; FF: fertilizer combined with fungicide; PE7: Broth culture of *B. subtilis* PE7. The data were presented as the mean ± standard deviation of six replicates. Different letters in the same column indicate significant differences between treatments, as determined by the least significant difference (LSD) test at a *p* < 0.05 significance level.

	Length (cm)		Fresh Weight (g)		Dry Weight (g)		Leaf Number	Leaf Area (cm^2^)	Strain PE7 in Pot Soil
	Shoot	Root	Shoot	Root	Shoot	Root			
Con	86.02 ± 1.22 ^b^	21.40 ± 1.76 ^a^	73.52 ± 3.78 ^b^	13.77 ± 2.56 ^b^	8.14 ± 0.52 ^b^	1.17 ± 0.21 ^b^	22.67 ± 0.52 ^b^	1111.83 ± 35.76 ^c^	-
FF	82.72 ± 5.65 ^b^	23.72 ± 0.79 ^a^	77.06 ± 9.06 ^b^	15.31 ± 3.83 ^ab^	7.90 ± 0.69 ^b^	1.16 ± 0.26 ^b^	24.33 ± 1.97 ^a^	1269.07 ± 12.36 ^b^	-
PE7	108.87 ± 5.17 ^a^	24.98 ± 1.71 ^a^	94.22 ± 4.23 ^a^	19.62 ± 4.21 ^a^	9.41 ± 0.25 ^a^	1.54 ± 0.40 ^a^	24.83 ± 0.98 ^a^	1388.66 ± 96.14 ^a^	1.73 ± 0.34 × 10^7^ CFU mL^−1^

**Table 2:** Effect of different treatments on the nutrient content of melon plants. (Con: fertilizer only; FF: fertilizer combined with fungicide; PE7: Broth culture of *B. subtilis* PE7). Different letters in the same column indicate significant differences between treatments, as determined by the least significant difference (LSD) test at a *p* < 0.05 significance level.

**Treatment**	**Macronutrient (mg plant^−1^)**					
	**N**	**P**	**K**	**Mg**	**Ca**	
Con	132.84 ± 5.37 ^b^	11.57 ± 0.57 ^a^	416.66 ± 8.98 ^b^	83.98 ± 8.52 ^a^	284.83 ± 12.86 ^b^	
FF	131.94 ± 5.47 ^b^	11.46 ± 1.17 ^a^	416.66 ± 8.98 ^b^	86.75 ± 7.66 ^a^	340.66 ± 14.57 ^a^	
PE7	199.23 ± 7.92 ^a^	11.13 ± 0.92 ^a^	488.27 ± 20.50 ^a^	98.76 ± 10.63 ^a^	328.86 ± 16.50 ^a^	
**Treatment**	**Micronutrient (mg plant^−1^)**					
	**Mn**	**B**	**Cu**	**Fe**	**Mo**	**Zn**
Con	3.56 ± 0.20 ^b^	171.60 ± 1.73 ^b^	962.57 ± 275.3 ^a^	31.38 ± 11.27 ^a^	7.25 ± 0.51 ^a^	0.24 ± 0.03 ^c^
FF	3.78 ± 0.11 ^b^	162.85 ± 2.41 ^b^	1275.7 ± 227.5 ^a^	29.01 ± 5.63 ^a^	6.43 ± 0.51 ^a^	0.34 ± 0.01 ^b^
PE7	7.56 ± 0.59 ^a^	344.34 ± 24.53 ^a^	1450.4 ± 322.1 ^a^	30.82 ± 13.03 ^a^	4.07 ± 0.98 ^b^	0.50 ± 0.06 ^a^

N, nitrogen; P, phosphorous; K, potassium; Mg, Magnesium; Ca, Calcium; Mn, Manganese; B, Boron; Cu, Copper; Fe, Iron; Mo, Molybdenum; Zn, Zinc.

## Data Availability

All the data are available within the manuscript.
